# Long-term effects of incubation temperature on growth and thermal physiology in a small ectotherm

**DOI:** 10.1098/rstb.2022.0137

**Published:** 2023-08-28

**Authors:** Madeleine J. De Jong, Lesley A. Alton, Craig R. White, Moira K. O'Bryan, David G. Chapple, Bob B. M. Wong

**Affiliations:** ^1^ School of Biological Sciences, Monash University, Melbourne, Victoria 3800, Australia; ^2^ Centre for Geometric Biology, School of Biological Sciences, Monash University, Melbourne, Victoria 3800, Australia; ^3^ School of BioSciences and BIO21 Institute, University of Melbourne, Parkville, Victoria 3010, Australia

**Keywords:** phenotypic plasticity, developmental temperature, growth rate, thermal performance, metabolic rate, sperm

## Abstract

Thermal conditions in the developmental environment can substantially affect an individual's phenotype, particularly in egg-laying ectotherms. However, whether these effects persist into adulthood is rarely examined. To investigate this, we incubated delicate skink, *Lampropholis delicata*, eggs at either cool (22°C), mild (26°C) or hot (30°C) temperatures. After hatching, we measured growth, thermal performance curves of locomotor activity, and thermal sensitivity of resting metabolic rate of offspring as juveniles (4–6 weeks of age), sub-adults (approx. 200 days of age), and adults (approx. 2 years of age), and then measured developmental temperature impacts on male fertility. Incubation temperature had a lasting effect on growth and locomotor performance, with cool and hot incubation temperatures resulting in faster growth and larger maximum size, and hot incubation temperatures reducing locomotor performance at all timepoints. Effects on resting metabolic rate were only present in sub-adults, with a higher metabolic rate at high and average body mass and negative metabolic scaling exponent in cool-incubated lizards. Additionally, cool and hot incubation treatments resulted in shorter sperm midpieces and heads. Incubation temperature did not affect testis mass or sperm count. Overall, our results demonstrate that incubation temperature can have lasting effects on later life stages, highlighting the importance of maternal nest-site selection, but that some effects are age dependent.

This article is part of the theme issue ‘The evolutionary ecology of nests: a cross-taxon approach’.

## 1. Introduction

The phenotypes of individuals can change in response to environmental variation, a process known as phenotypic plasticity [1,2], with developmental environments known to have a strong impact [3,4]. Mothers can, therefore, play an important role in determining offspring fitness by influencing developmental environment; for example, maternal allocation of hormones or resources, or, for egg-laying species, judicious nest-site selection [5,6]. Nest temperature is vital for offspring success, influencing survival probabilities and controlling many biochemical processes which can affect, particularly in ectotherms, developmental processes such as metabolic and growth rates [7,8], as well as a broad range of post-hatching phenotypic traits [9]. Understanding how these phenotypes change in response to differences in nest temperature is vital in gaining insight into the effects of anthropogenic warming, one of the biggest challenges animals face globally.

The effects of nest temperature on phenotype can persist into adulthood, emerge later in life or even be strengthened with age [3,4,9,10]. For example, in Yucatan banded geckos, *Coleonyx elegans*, a high incubation temperature decreased activity and antipredator behaviour in adults 450 days post-hatching [11]; in Brisbane river turtles, *Emydura signata*, mass and swimming performance were affected by incubation temperature 1 year post-hatching [12]; and in bearded dragons, *Pogona vitticepts*, 1-year-olds that had been incubated at a cold temperature were faster at completing a social learning task [13]. Even traits that do not mature until adulthood can be affected. Male fertility, in particular, is highly sensitive to temperature in many species, and to a greater extent than female fertility [14], and incubation temperature has been demonstrated to impact male reproductive success in jacky dragons, *Amphibolurus muricatus* [15]. However, it is unknown whether this can occur as a direct result of egg incubation temperature effects on fertility or indirectly via effects on other organs or systems, such as body size and metabolic rate, which are linked to reproductive output [16].

Effects of variation in temperature during development are theorized to persist into later life stages due to several possible, non-mutually exclusive reasons. For example, as animals grow along a particular developmental trajectory, they may become more constrained in what phenotype they can express, or it may be more costly to adjust to an alternative phenotype [4,17]. However, this might not always be the case. There can be additional times throughout life outside of the early developmental environment where the potential for plasticity is increased (i.e. ‘sensitive windows of development’), such as the onset of reproduction [17]. Alternatively, effects of developmental environments, such as nest temperature, may weaken over the lifespan, which was found in a meta-analysis investigating effects of developmental stressors on a broad range of species [18], or be age-dependent, such as in bearded dragons where incubation temperature affected boldness behaviour differently at different ages [19]. The extent to which these effects persist may influence the impact of warmer nest temperatures associated with climate change. Long-term studies are, however, still relatively rare ([9,20], although see [13,19,21]), and, therefore, it is difficult to generalize as to whether such nest temperature effects persist or diminish with age, or otherwise emerge at different life stages as has been observed in some turtles (e.g. Brisbane river turtle: [12]).

Within this study, we investigated whether variation in nest temperature had long-term effects on the phenotypes of ectotherms, using a model lizard species, the delicate skink, *Lampropholis delicata*. This species exhibits variation in thermal physiology [22,23], as well as developmental plasticity in behaviour and locomotor performance in response to variation in incubation temperatures [24,25], although the persistence of these effects beyond early life remains unknown. To address this question, we incubated eggs across three incubation treatments, at cool (22°C), mild (26°C) and hot (30°C) temperatures, and measured resting metabolic rate (RMR), body mass and sprint speed in skinks as juveniles, sub-adults and adults. We also measured fertility in adult skinks, but, due to logistical constraints in measuring fertility traits in females, restricted this to males, measuring testis mass as well as sperm quantity and quality in adult males. From these data, we estimated growth rates using von Bertalanffy curves [26] and thermal performance curves from sprint speed to determine: (i) whether effects of incubation temperature differ in their presence or absence depending on lizard age; and (ii) whether incubation temperature affects traits that are only measurable at maturity, i.e. male fertility. Knowledge of the long-term effects of nest temperature in ectotherms will provide insights into developmental plasticity and how reptiles may be affected by anthropogenic climate warming.

## 2. Methods

### (a) Animal collection and husbandry

Delicate skinks are small (34–55 cm snout–vent length [SVL]), oviparous lizards that inhabit the east coast of Australia [27]. In the early breeding season (12–27 September) of 2018, gravid delicate skinks were collected from urban parks in Sydney (33°47′ S–33°54′ S, 151°03′ E–151°14′ E) and transported to Monash University (Melbourne, Australia). Lizards were collected using hand capture and mealworm fishing methods, which do not result in a phenotype capture bias [28]. Lizards were housed individually in plastic containers (25 × 20 × 18 cm) in temperature-controlled rooms (approx. 22°C), with lighting (supplemented with UV light) on between 06.00 and 20.00 h. Each container was lined with newspaper and contained a dish with a moist soil substrate, with heat mats underneath the containers creating a thermal gradient (21–34°C), available 24 h, to allow lizards to behaviourally thermoregulate. Skinks were fed crickets, *Acheta domesticus*, dusted in vitamin powder three times per week and had water available ad libitum. We checked the containers twice daily, in the morning and afternoon, to collect eggs within 14 h of laying.

### (b) Experimental design

Immediately after collection, we weighed each egg to the nearest 0.0001 g, and then placed it into a small plastic container with moist vermiculite (1 : 1.2 vermiculite to water ratio by mass: [25]). The containers were covered to prevent water loss and each clutch was split randomly across three incubators (IC24, Labec, Marrickville, NSW, Australia) set to different temperatures. Delicate skink clutches are typically between three and four eggs [29], although in our study clutches ranged between one and three eggs and each treatment therefore did not have more than one egg from each clutch. Our mild incubation treatment, 26 ± 3°C, was based on natural nest temperatures recorded at an urban park in Sydney (33°53′ S 151°10′ E) [30]. A temperature of 30 ± 3°C was used for the hot incubation treatment, to simulate a rise of 2°C above average temperatures recorded in warmer urban nests (28 ± 3°C) in the same Sydney park [24]. This represents 2°C of climate warming, which is predicted to occur in Sydney by 2050 [31]. Finally, our cool incubation treatment averaged 22 ± 3°C, which is 4°C below the mild treatment and falls within the range of present-day temperatures of shaded natural nests [24]. Eggs were rotated between incubator shelves twice per week to minimize potential effects of thermal gradients within the incubators and eggs were checked daily for signs of hatching. Once hatched, we weighed the hatchlings to the nearest 0.0001 g and housed them individually in identical conditions as their parents for the duration of the experiment. The housing containers (25 × 20 × 18 cm) were lined with newspaper and contained hen egg carton as shelter, had 24 h access to a thermal gradient (21–34°C) created with heat mats, and lighting supplemented with UV between 6:00 and 20:00 h. Hatchlings had water available ad libitum and were fed baby crickets dusted in vitamin powder three times weekly.

Resting metabolic rate, sprint speed and body size (mass, g) were measured at three different timepoints over 2 years. We first measured the skinks when they were juveniles of between four and six weeks of age. Due to differences in incubation duration, laying dates and, therefore, hatching dates (spanning approx. four months from 26 November 2018 to 25 March 2019), experiments for juveniles were staggered based on age. Traits were measured a second time as sub-adults (August to October 2019; average age: 209 days), beginning just prior to the onset of the reproductive season in Sydney [29], and then a third time as adults (November to December 2020; average age: 655 days). Staggering of experiments with treatment groups matched based on age and mass has been done previously [19,21], demonstrating in bearded dragons that differences among treatment groups that did not overlap in age in sprint performance, but not foraging behaviour, depended on whether groups were matched for age or mass [21]. In our study, as by the time delicate skinks reached sub-adulthood there were minimal differences in mass, as well as overlap in ages among treatments, we did not stagger experiments based on age for sub-adults or adults. Skinks were sexed once they matured (SVL > 30 mm) by everting the hemipenes after the sub-adult experiments. Males were measured for sperm quality and quantity at the conclusion of the experiment (see below), at which point final survival of skinks was recorded, approximately four months after adult phenotype measurements.

### (c) Sprint speed and thermal performance curves

We measured sprint speed at five different temperatures (15, 20, 25, 30 and 35°C) in a random order, following established methods [22,23]. Skinks were tested at each temperature three times on the same day, and were placed into thermal chambers heated or cooled to the test temperature for 30 min prior to, and between, each run to equilibrate body temperature with the test temperature. Where skinks were measured for multiple temperatures on a single day, they were rested for a minimum 2 h between temperatures [32]. Food was withheld for 24 h prior to measurement to standardize digestion levels. We raced skinks individually down a 1 m racetrack marked into four 25 cm segments and set to the test temperature, encouraging sprinting by tapping the tail of the lizard with a paintbrush. Runs were recorded with a high-speed camera at 30 frames per second (Casio EX- ZR50), and we used video playback in BORIS [33] to record the time to run through each 25 cm segment. The fastest time from each temperature (across the three runs) was used as a measure of sprint speed (*V*_max_: [22]). We then used *V*_max_ to estimate thermal performance curves (TPC) with rTPC [34], fitting curves with a Gaussian distribution [35]. Curves were bounded at critical thermal minima (4.7°C) and maxima (40.8°C), which were based on published data [36]. To assess differences between incubation treatments in TPC shape, we then extracted optimal performance temperature (*T*_opt_: the temperature where an individual attains maximum speed, i.e. the location of the peak of the TPC along a temperature gradient), predicted maximum speed (*P*_max_: speed at the optimal temperature, i.e. height of the TPC), and performance breadth (*P*_80_: breadth of temperatures where an individual sprints at 80% of *P*_max_, i.e. width of the TPC).

### (d) Resting metabolic rate

The resting metabolic rate (RMR) of individual skinks were measured during the day at three temperatures (15, 25 and 35°C) in a random order at each timepoint. Prior to metabolic rate measurements, we withheld food from the skinks for two days to standardize digestion [37], and we weighed skinks immediately prior to measurements. The rate of CO_2_ production (ml h^−1^) was measured as a proxy for metabolic rate using an eight-channel respirometry system. The respirometry system was supplied with room air that was pumped through columns of soda lime and Drierite to remove CO_2_ and water vapour, respectively. The flow rate of dry, CO_2_-free air through each of the eight channels of the respirometry system was regulated nominally to 60 ml min^−1^ through a mass flow controller (Aalborg, Model GFC17, Orangeburg, NY, USA). The volumetric flow rates produced by the flow controllers was measured using a Gilian Gilibrator-2 NIOSH Primary Standard Air Flow Calibrator with a low-flow cell (Sensidyne LP, St Petersburg, FL, USA) and corrected to STPD (i.e. 101.325 kPa and 0°C). After the flow controller, air flowed through a glass respirometry chamber containing a skink that was housed inside a controlled-temperature cabinet set to the nominal test temperature. The fractional CO_2_ concentration of the excurrent air ($F_{{\mathrm{eCO}}_{2}}$) from the respirometry chamber was measured for 80 min at a frequency of 1 Hz with a CO_2_/H_2_O gas analyser (LI-COR, Model LI-840, Lincoln, NE, USA) calibrated with precision span gases (11.4 ppm, 30.4 ppm and 50.3 ppm for juveniles and 30.4 ppm, 169.7 ppm and 200.1 ppm for adults). The fractional CO_2_ concentration of the incurrent air into an empty chamber was measured for 7 min before and after the skink was placed inside the chamber and a linear model was fitted to this data to estimate the fractional CO_2_ concentration of the incurrent air during the 80 min measurement period ($F_{{\mathrm{iCO}}_{2}}$). The rate of CO_2_ production of the skink over the 80 min measurement period was calculated by subtracting $F_{{\mathrm{iCO}}_{2}}$ from $F_{{\mathrm{eCO}}_{2}}$ and multiplying by the STPD-corrected flow rate. The first 25 min of data were discarded to allow the skink to recover from handling stress, and the lowest rate of CO_2_ production over 5 min was selected as the measure of resting metabolic rate.

### (e) Male fertility

In April 2021, we humanely killed males via intraperitoneal injection of 0.1 ml of 0.1 ml sodium pentabarbitone that was diluted to 1 ml. We then dissected the male skinks and weighed each testis to the nearest 0.0001 g. Immediately after dissection, one testis per individual was placed into 600 ml of Triton X-100 solution (0.15 M NaCl, 0.1 mM NaN_3_, 0.05% Triton X-100) and set aside for sperm counts. We also immediately placed one epididymis per individual into 200 µl of Ham's F-10 medium (Irvine Scientific, Santa Ana, California, USA: [38–40]) and sliced it into thirds to allow sperm to swim out. After 10 mins, an aliquot of solution was pipetted onto a slide, fixed in 10% buffered formaldehyde solution and stained with haematoxylin and eosin. Images of the sperm were captured using a BX53F2 Olympus microscope (Tokyo, Japan). The lengths of a minimum of 90 sperm heads, midpieces, and tails were measured for each male using a microscope and Fiji imaging software [41]. To measure sperm counts, the testes were sonicated in the Triton X-100 solution to destroy all non-condensed germ cell nuclei (i.e. only condensed elongated sperm heads remained: [42]). The number of sperm per testis was then determined using a hemocytometer.

### (f) Statistical methods

We analysed data in R v. 4.1.2 [43]. Bayesian mixed-effects models were used to model growth curve parameters (*K*, growth constant; *L*_inf_, asymptote; and *t*_0_, theoretical age at body size 0) using von Bertalanffy curves [26] and determine the effects of incubation treatment and age class on phenotype using *brm* (package brms: [44]). All models were run with four chains with a minimum of 10 000 iterations (3000 warmup) using weakly informative priors. To facilitate interpretation of fixed effects, we mean-centred (mean = 0, s.d. = 1) continuous covariates prior to analysis. To investigate effects of incubation treatment on survival to hatching and the end of the experiment, we fitted Bernoulli models with survival as a binomial response variable and incubation treatment as a fixed effect. To determine whether hatchling mass varied with incubation treatment, we fitted models with Gaussian error distributions and incubation treatment and egg mass as fixed effects. To examine if there were age-dependent effects of incubation treatment on phenotype, fixed effects of mass (centred), age class (juvenile, sub-adult, adult), incubation treatment and an age class by incubation treatment interaction were included in all models. All models also included individual ID and maternal ID as random intercepts to account for repeated measures and relatedness between individuals, respectively. Where a trait was measured at multiple temperatures (RMR and sprint speed), we also included measurement temperature as a fixed effect. Finally, to investigate effects of incubation treatment on male fertility, we fit sperm length and testis mass models with Gaussian distributions and sperm counts with a lognormal distribution. These models contained incubation treatment as fixed effects and maternal ID as a random effect. Testis mass was also included as a covariate in analysis of sperm counts, mass for analysis of both testis mass and sperm counts, and total sperm length was square root transformed to facilitate model convergence. We checked for model convergence using trace plots, and that *R*_hat_ values = 1. Where credibility intervals (CrIs) of an estimate of an effect or a pairwise comparison among treatments did not overlap 0 (i.e. both estimates were either positive or negative), we interpreted the estimate as significant. *Post-hoc* tests were performed using *emmeans* and *emtrends* (package emmeans: [45]). Estimates of posterior means and emmeans contrasts are reported with 89% CrIs [46].

## 3. Results

### (a) Survival and hatchling mass

A total of 73 eggs from 34 clutches were incubated (21 in the cool treatment, 25 in the mild and 27 in the hot). Of these, 54 hatched successfully with a hatching success of 66.7% (*n* = 14) in the cool treatment, 84.0% (*n* = 21) in the mild treatment and 70.4% (*n* = 19) in the hot treatment. Incubation temperature did not significantly affect survival to hatching (CrIs overlapped 0). Of those that survived to hatching, 40 skinks had survived to maturity and could be sexed (10 from the cool treatment [males: *n* = 5, females: *n* = 5], 15 from the mild treatment [males: *n* = 9, females: *n* = 6], and 15 from the hot treatment [males: *n* = 5, females: *n* = 10]) and 34 skinks survived to the end of the experiment (10 from the cool treatment [males: *n* = 5, females: *n* = 5], 11 from the mild treatment [males: *n* = 9, females: *n* = 2], and 13 from the hot treatment [males: *n* = 5, females: *n* = 8]). There was no significant effect of incubation treatment on survival to maturity (CrIs overlapped 0), sex ratios at maturity (CrIs overlapped 0), or survival from hatching to the end of the experiment (CrIs overlapped 0). Incubation temperature did, however, affect hatching mass, with hot-incubated skinks lighter on hatching (estimated marginal mean of posterior distribution, estimate [89% CrI]: *β*_Hot_ = 0.10 [0.09, 0.10]) than skinks from both the mild (*β*_Mild_ = 0.11 [0.10, 0.11]) and cool (*β*_Cool_ = 0.11 [0.11, 0.11]) incubation treatments (pairwise contrasts [89% CrI]: cool-mild = −0.003 [−0.008, 0.003]; cool-hot = 0.008 [0.001, 0.014]; mild-hot = 0.010 [0.005, 0.016]).

### (b) Sprint speed and thermal performance curves

**Figure 1.**
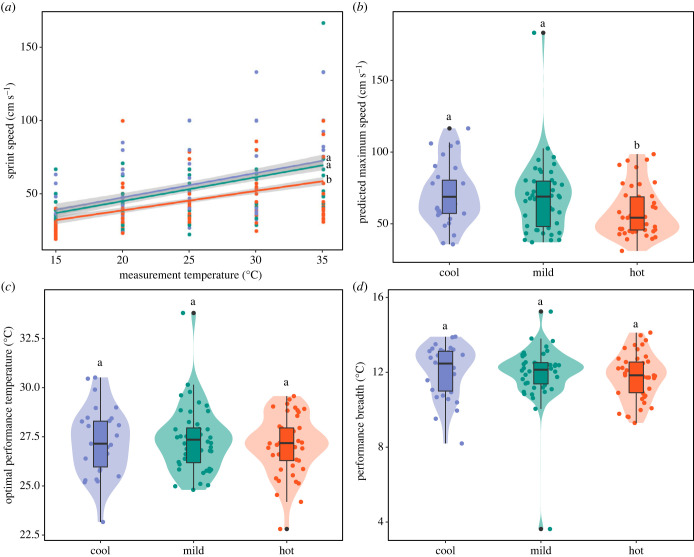
Effects of incubation temperature (cool, blue; mild, green; hot, red) on sprint speed (*a*) and thermal performance curve parameters (predicted maximum speed (*b*); optimal performance temperature (*c*); and performance breadth (*d*)). Sprint speed (*a*) is shown across five measurement temperatures, with grey shading indicating 89% confidence intervals. Violin and boxplots (*b–d*) show medians, interquartile ranges and 1.5× interquartile ranges (whiskers). Dots correspond to single measurements for each skink. Lower case letters show which groups are significantly different from one another, with significant differences between groups indicated by different lower case letters. (Online version in colour.)

Effects of incubation treatment on sprint speed did not change with age, with no interaction between age and incubation temperature (CrIs overlapped 0; figure 1*a*). There was, however, a main effect of incubation temperature, with hot-incubated skinks being slower sprinters (estimated marginal mean of posterior distribution, estimate [89% CrI]: *β*_Hot_ = 3.77 [3.69, 3.84]) than those from the mild (*β*_Mild_ = 3.90 [3.83, 3.98]) and cool (*β*_Cool_ = 3.95 [3.86, 4.04]) treatments (pairwise contrasts [89% CrI]: cool-mild = 0.04 [−0.06, 0.15]; cool-hot = 0.18 [0.07, 0.29]; mild-hot = 0.14 [0.04, 0.23]). Additionally, effects of measurement temperature on sprint speed changed with age. The increase in sprint speed with an increase in temperature was smallest in juvenile skinks (measurement temperature × age interaction, slope estimate [89% CrI]: *β*_TempJuvenile_ = 0.02 [0.02, 0.02]) compared to sub-adults (*β*_TempSubadult_ = 0.03 [0.03, 0.04]) and adults (*β*_TempAdult_ = 0.04 [0.03, 0.04]).

We found no effect of the interaction between incubation treatment and age on the shape of the thermal performance curve, as indicated by the parameters extracted from the curve (CrIs overlapped 0; figure 1*b–d*). There was, however, a significant effect of mass (slope estimate [89% CrI]: *β*_Mass_ = 12.23 [7.20, 17.33]) and main effect of incubation treatment on predicted maximum speed (figure 1*b*). Hot-incubated skinks had a slower predicted maximum speed (estimated marginal mean of posterior distribution, estimate [89% CrI]: *β*_Hot_ = 57.0 [51.8, 62.1]) than skinks incubated in both cool (*β*_Cool_ = 66.9 [60.5, 73.0]) and mild (*β*_Mild_ = 64.7 [59.3, 70.0]) treatments (pairwise contrasts [89% CrI]: cool-mild = 2.25 [−5.14, 8.92]; cool-hot = 9.91 [2.59, 17.33]; mild-hot = 7.63 [1.13, 13.94]). By contrast, while mass had a positive (slope estimate [89% CrI]: *β*_Mass_ = 0.04 [0.01, 0.06]) and negative (*β*_Mass_ = −1.18 [−1.84, −0.50]) effect on optimal performance temperature and performance breadth, respectively, neither measure was significantly affected by incubation treatment (CrIs overlapped 0; figure 1*c,d*).

### (c) Resting metabolic rate

**Figure 2.**
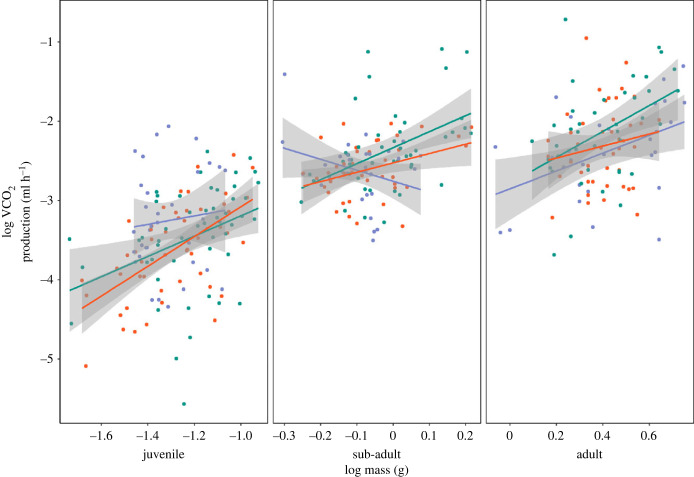
Effects of incubation treatment (cool, blue; mild, green; hot, red) on resting metabolic rate. Coloured points represent measurements of individual skinks and grey shading indicates 89% confidence intervals. CO_2_ production was standardized across measurement temperatures using a Q_10_ of 2.0 [47] with the equation $\mathrm{MR}2=\mathrm{MR}1\times Q_{10}^{\left[T2-T1/10\right]}$.(Online version in colour.)

Effects of incubation treatment on RMR differed depending on the age and mass of the skinks (figure 2). As juveniles and adults, there were few significant differences among incubation treatments (figure 2). However, as sub-adults, skinks incubated at a cool temperature had a higher metabolic rate at both average and low mass, but a lower metabolic rate at a high mass compared to both hot and mild incubation treatments (figure 2, electronic supplementary material, table S1).

There were also differences among treatments in the mass scaling exponent, which were only present in sub-adults (CrIs overlapped 0; figure 2, middle panel). The mass scaling exponent was negative in sub-adult cool-incubated skinks (mass × incubation × age interaction, slope estimate [89% CrI]: *β*_MassCoolSubadult_ = −0.60 [−1.78, 0.47]) whereas it was positive for mild- (*β*_MassMildSubadult_ = 1.81 [1.08, 2.54]) and hot-incubated (*β*_MassHotSubadult_ = 1.27 [0.45, 2.15]) skinks (pairwise contrasts: cool-mild = −2.40 [−3.69, −1.07]; cool-hot = −1.86 [−3.24, −0.48]; mild-hot = 0.55 [−0.57, 1.63]).

### (d) Growth rate

**Figure 3.**
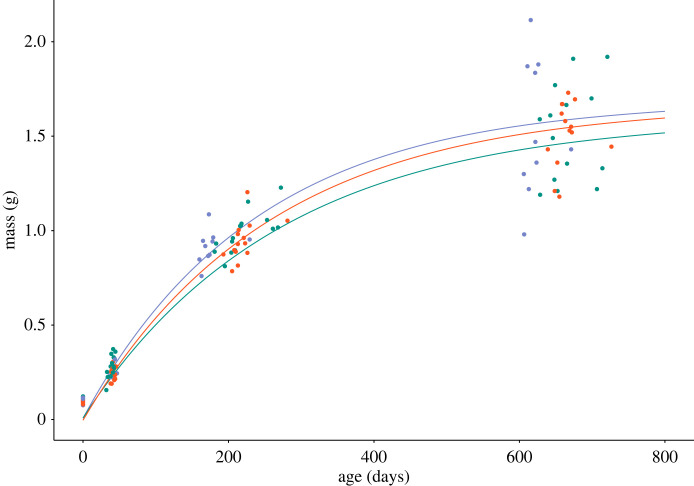
Von Bertalanffy growth curves for delicate skinks incubated at a cool (blue), mild (green) or hot (red) temperature derived from results of a Bayesian mixed effects model of growth. Coloured points represent measurements of mass for individual skinks. (Online version in colour.)

The growth constant, *K*, was significantly affected by incubation treatment, with mild-incubated skinks having the lowest growth constant (estimated marginal mean of posterior distribution, estimate [89% CrI]: *β*_Mild_ = 0.0037 [0.0034, 4.8000]) compared to both cool- (*β*_Cool_ = 0.0042 [0.0038, 7.25]) and hot- (*β*_Hot_ = 0.0039 [0.0036, 10.21]) incubated skinks (pairwise contrasts: cool-mild = 0.0006 [0.0002, 2.4500]; cool-hot = 0.0002 [0.0008, −2.9500]; mild-hot = −0.0002 [−5.4000, −0.00003]). Similarly, *L*_inf_ was lowest in mild-incubated skinks (*β*_Mild_ = 1.60 [0.91, 1.69]) compared to cool- (*β*_Cool_ = 1.69 [1.61, 1.84]) and hot- (*β*_Hot_ = 1.67 [1.32, 1.77]) incubated skinks (pairwise contrasts [89% CrI]: cool-mild = 0.13 [0.03, 0.71]; cool-hot = 0.06 [−0.06, 0.30]; mild-hot = −0.09 [−0.40, −0.02]). By contrast, *t*_0_ did not differ significantly among incubation treatments (all CrIs overlapped 0; figure 3).

### (e) Male fertility

Incubation temperature did not have any significant effect on testis mass or sperm counts (all CrIs overlapped 0; table 1), although testis mass had a positive effect on sperm counts (slope estimate [89% CrI]: *β*_Testismass_ = 1.29 [0.48, 2.09]). By contrast, while total sperm length was not significantly affected by incubation temperature (CrIs overlapped 0), sperm midpieces of both cool (estimated marginal mean of posterior distribution, estimate [89% CrI]: *β*_Cool_ = 8.97 [8.48, 9.47]) and hot (*β*_Hot_ = 9.03 [8.54, 9.58]) treatments were shorter than mild-incubated skinks (*β*_Mild_ = 9.83 [9.39, 10.29]; pairwise contrasts [89% CrI]: cool-mild = −0.88 [−1.42, −0.22]; cool-hot = −0.06 [−0.79, 0.66]; mild-hot = 0.79 [0.11, 1.49]). Sperm head lengths of skinks incubated at a cool temperature (*β*_Cool_ = 4.31 [3.87, 4.75]) were also shorter than mild-incubated skinks (*β*_Mild_ = 4.90 [4.48, 5.30]; pairwise contrast [89% CrI]: cool-mild = −0.62 [−1.10, −0.05]), but there were no other differences between treatments in sperm head or tail length (CrIs overlapped 0).

**Table 1.** Measures of male fertility of delicate skinks incubated at cool, mild and hot temperatures (mean ± s.d.).

	cool incubation	mild incubation	hot incubation
testis mass (mg)	24.46 (± 11.98)	23.85 (± 10.67)	21.33 (± 5.33)
sperm count (×10^4^)	204 (± 443)	203 (± 385)	90 (± 171)
sperm length (µm)			
head	4.33 (± 0.54)	4.86 (± 0.40)	4.35 (± 0.50)
midpiece	9.04 (± 0.53)	9.78 (± 0.56)	9.04 (± 0.41)
tail	80.50 (± 2.53)	79.36 (± 3.50)	81.37 (± 2.37)
total length	93.87 (± 1.96)	94.00 (± 3.14)	94.76 (± 2.06)

## 4. Discussion

Our results demonstrate that incubation temperature has long-term effects on growth and physiology in the delicate skink. Moreover, these effects, where they were present at early ages, did not appear to diminish over time, and, in some cases, emerged later in life at adulthood. Growth rate, RMR and reproduction are interrelated [16], and, while primarily demonstrated in fish, growth can also affect locomotor performance through muscle development [48,49]. However, there were no clear patterns demonstrating this interrelatedness in response to variation in incubation temperatures. Both hot and cool incubation treatments had increased growth rates, yet only the cool treatment differed in RMR and only the hot treatment in sprint speed. While it is unclear why this is the case, developmental temperatures nevertheless have a long-term effect on delicate skink phenotype, highlighting the potential for climate warming to have significant impacts on reptile populations.

Incubation temperature had a long-term effect on sprint speed, with reduced running speed in hot-incubated skinks. This was also reflected in the predicted maximum speed parameter of the thermal performance curve, but optimal performance temperature and performance breadth did not differ among treatments. Incubation temperature effects on locomotor performance in lizards are highly variable. For example, garden skinks, *Lampropholis guichenoti*, also ran slower when incubated at a hotter temperature [30], but, in contrast, Suter's skink, *Oligosoma suteri*, ran slower when incubated at a cool temperature [50], while others displayed no effect, such as in jacky dragons [51]. Faster sprint speeds have been associated with higher survival probabilities in hatchling, but not adult, collared lizards, *Crotaphytus collaris*, in a field-based study [52]. With an increase in average temperatures experienced during development of 4°C, climate change may result in reduced survival probabilities of delicate skinks, at least until reaching maturity. However, we found no variation among incubation treatments in survival. It is possible that effects of sprint performance on survival are more prevalent in the wild, as in collared lizards [52], and that we did not observe these effects because our study on delicate skinks was conducted in captivity. Differences in sprint performance may arise through effects of developmental temperature on morphology or physiology, such as limb length or musculature. Variation in growth rates in fish due to developmental dietary or temperature manipulation can influence growth of muscle mass, and therefore affect swimming performance [48,49]. Interestingly, only skinks from the hot, and not cool, incubation treatment had reduced sprint speed, despite not differing significantly in growth rates. This may be due to differences in hatchling mass and final body size, with cool-incubated skinks heavier in both instances, or, alternatively, incubation temperature may have affected limb length, indirectly affecting sprint performance. This occurred in closed-litter rainbow skinks, *Carlia longipes*, where cool incubation temperatures resulted in longer-limbed skinks that were faster sprinters [53].

We also found effects of incubation treatment on RMR in delicate skinks, but only in sub-adults. There are few studies investigating effects of thermal developmental plasticity on RMR in reptiles, and results, similar to those investigating other traits, vary in the direction and presence of an effect. In snapping turtles, *Chelydra serpentina*, both cooler [54] and intermediate [55] incubation temperatures increased metabolic rates, but others have found no effect, demonstrated in the African spurred tortoise, *Geochelone sulcata* [56], and in delicate skinks at approximately 2.5 months of age [57]. As higher metabolic rates can increase growth through increased energy assimilation [58], we might expect an observable difference in RMR when differences in growth rates are at their greatest. If growth rates were highest between delicate skinks at the sub-adult stage, this may explain the variation in developmental temperature effects on CO_2_ production with age we observed in delicate skinks. As sub-adults, cool-incubated skinks also had a shallower mass scaling exponent, indicating a greater energetic efficiency with increasing body size [59], which may have enabled faster growth rates. However, hot-incubated skinks also had faster growth rates, but did not exhibit a difference in RMR. As body mass significantly affects RMR, as well as subsequent growth rates, as in jacky dragons [51], the differences we observed between hot and cool incubation treatments in initial hatchling size, and the potential resulting impact on subsequent growth, may help explain the variation in patterns of growth and RMR we observed in delicate skinks.

It is well-known that growth rates are affected by developmental temperatures [9], with higher and lower incubation temperatures in different species of lizard increasing post-hatching growth rates [60]. Here, we found that both hot and cool incubation temperatures increased the growth rate constant in delicate skinks, as well as the maximum body size. Faster growth rates may allow individuals hatching with either a smaller body size (from a hot incubation temperature) or a delayed hatching time (from a cool incubation temperature) to ‘catch up’ to other individuals, provided environmental conditions have improved. Specifically, for the hot incubation treatment, this may compensate for climate change reducing initial body size. High growth rates can, however, be costly at the cellular and ecological levels [10], leading to, for example, reduced lifespan (e.g. three-spined sticklebacks, *Gasterosteus aculeatus* [61]) and higher predation though increased foraging effort (e.g. speckled wood butterfly, *Pararge aegeria*, larvae [62]). Whether there is a significant trade-off to higher growth, or even the ability to achieve higher growth rates, will depend on resource availability and the intensity of competition in the post-hatching environment, which is also expected to be altered by climate change.

Both cool- and hot-incubated skinks had shorter sperm midpieces and heads than skinks incubated in the mild treatment, although total sperm length did not differ. Previous studies have found increased developmental temperatures decreased sperm length in Indian meal moths, *Plodia interpunctella* [63] and in Trinidadian guppies, *Poecilia reticulata* [64]. Sperm length can affect other aspects of sperm quality, such as velocity, which also decreased in Trinidadian guppies that developed at a high temperature [64], and have a negative relationship with sperm longevity [65]. Specifically, in house mice, *Mus musculus*, sperm velocity was correlated with midpiece length [65]. Both increasing and decreasing incubation temperatures may therefore impact sperm swimming performance and longevity in delicate skinks, affecting post-copulatory competition among males. It is possible that the observed differences in sperm length are the result of a trade-off with other life-history traits, such as growth rate, given the simultaneous increase in growth rates and decrease in sperm midpiece and head lengths of cool- and hot-incubated male delicate skinks. By contrast, testis mass and sperm counts were unaffected by incubation temperature, despite effects demonstrated in other species (e.g. cowpea seed beetles, *Callosobruchus maculatus*: [66]). As food was not limited and intra-specific competition was absent in captivity, trade-offs between life-history traits may not have greatly constrained investment into reproduction in male delicate skinks, hence the observed changes in only some sperm length measures. As climate warming progresses, other biotic and abiotic pressures may be altered, such as intra-specific competition [67], which might compound effects of climate warming on fertility. Alternatively, some aspects of sperm quality may be robust to variation in developmental temperature. While our results suggest impacts of incubation treatment on some male fertility traits in lizards, impacts on adult female reproductive success will also be vital in assessing the degree of climate change effects and needs further attention.

It has long been recognized that developmental environments can have persistent effects, and, while evidence for this in response to reptile incubation temperatures has been limited to date, those investigating long-term impacts have found lasting effects in adults [9,20]. Likewise, we found effects of incubation temperature persisted in delicate skinks close to 2 years of age. Organisms are expected to become less plastic in response to environmental change with age [4,17], whether this is due to developmental effects that are difficult to reverse later, or to altered gene expression, such as DNA methylation [68]. Regardless of mechanism, long-term effects of developmental temperatures in egg-laying species highlights the importance of maternal effects by way of nest temperatures for offspring. Nest sites are often selected based on microhabitat characteristics that correlate with temperatures that maximize fitness in offspring [69,70]. However, these characteristics can become decoupled from temperature in highly modified habitats, leading to suboptimal nest-site selection (e.g. in snapping turtles: [69]), which may compound effects of climate warming. Furthermore, while nest-site selection may be able to buffer offspring against rising temperatures from climate change, studies have found plasticity may only partially compensate for higher temperatures (e.g. eastern three-lined skinks: [71]), or be maladaptive, with reduced nest depth demonstrated in eastern fence lizards, *Sceloporus tristichus* [72]. If these responses are widespread, increasing our understanding of how incubation temperature effects persist in the long-term and correlate with fitness is increasingly urgent.

## 5. Conclusion

Overall, our study demonstrates long-term effects of incubation temperature on delicate skink phenotypes, highlighting the lasting impacts of maternal nest-site selection. Long-term studies are able to assess whether variation in developmental environments has implications for phenotype and fitness in adults as well as juveniles, thus influencing not only survival probability early in life, but also potential reproductive output and adult survival. An essential next step is to assess whether long-term effects are also present in more natural environments, as the post-hatching environment can also have a considerable influence on phenotype [51]. The characteristics of the post-hatching environment can itself be influenced by incubation temperature via hatching dates, as can the responses of individuals to environmental change [73], and teasing apart these subtle and indirect effects of incubation temperature will yield interesting and important insights into plasticity and, crucially, into how reptiles and other ectotherms may potentially be affected by anthropogenic change.

## Data Availability

Data for this study are available from the Figshare Repository: https://doi.org/10.26180/22211590.v1. The data are provided in electronic supplementary material [74].
